# Blackwater Fever in Children, Burundi

**DOI:** 10.3201/eid1107.041237

**Published:** 2005-07

**Authors:** Federico Gobbi, Sabrina Audagnotto, Laura Trentini, Innocent Nkurunziza, Manuel Corachan, Giovanni Di Perri

**Affiliations:** * Clinica Universitaria Malattie Infettive, Turin, Italy;; † Hopital de Kiremba, Ngozi, Burundi;; ‡ University Hospital, Barcelona, Spain

**Keywords:** blackwater fever, P. falciparum malaria, quinine, children

## Abstract

Blackwater fever is characterized by acute intravascular hemolysis with hemoglobinuria in patients with *Plasmodium falciparum* malaria. Its pathogenesis and management are still debated. Nine cases of this syndrome occurred in 2003 at Kiremba Hospital in Burundi in children receiving multiple quinine treatments.

Blackwater fever (BWF) is a clinical entity well known only in long-term residents in *Plasmodium falciparum*–endemic areas who take quinine irregularly. This syndrome became less frequent when chloroquine was the drug of choice for malaria from 1950 until the 1990s (1). Glucose-6-phosphate dehydrogenase (G6PD) deficiency is also frequently associated with the syndrome; however, its role is not determinant, as BWF is frequently described in patients with normal erythrocyte G6PD levels who are receiving quinine for severe malaria (2). Isolated cases have also been described with other antimalarials, such as halofantrine and mefloquine, which belong to the amino-alcohol drug family (3–5).

The pathogenesis of BWF thus remains unclear (4,6,7). Its management changed with the introduction of artemisinin derivates but is still debated. White and other researchers (2,8) state that parenteral quinine can be stopped when artemisinin derivatives are available because they seem to be safe and well tolerated.

Clinical features defining BWF are well established (2,9). The syndrome is characterized by severe intravascular hemolysis and anemia producing dark urine in patients with severe malaria. Abdominal pain, jaundice, hepatosplenomegaly, vomiting, and renal failure (especially in adults) have also been reported.

As *P. falciparum* resistance to chloroquine developed, quinine was increasingly used in clinical practice for treating intermittent malaria infections. BWF seemed to reappear at the end of the 1990s, according to descriptions in several European clinics of imported diseases (3–5). It particularly affected European missionaries with years of previous residence in malarious areas. In fact, some of the classical definitions of the syndrome described it in expatriate populations only (9). Cases of BWF in autochthonous populations have recently been described in the literature from Southeast Asia (10) and in African children in Senegal (7). We describe a large number of BWF cases in the pediatric ward of a hospital in the Burundi highlands, where no case has been observed in the previous 10-year period (1992–2002).

## The Study

Since January 1992, a hospital-based survey of malaria has been conducted at Kiremba Hospital in Ngozi Province. This 140-bed facility is located 1,540 m above sea level in the Burundi highlands; it serves a population of 75,000 (11).

For each case of malaria, laboratory data and clinical findings are recorded. Rising illness and death rates are being reported throughout Burundi, where *P. falciparum* accounts for most cases (12). According to the Kiremba Hospital registry, a 2-fold increase in admissions for malaria in the pediatric ward (children ≤14 years of age) was recorded from 1997 (658 cases) to 2002 (1,343 cases).

From February to December 2003, a period when 1,039 malaria patients were hospitalized, we observed 9 cases of severe intravascular hemolysis with dark urine in pediatric patients who had been treated with quinine. These children were all male with a mean age of 8.2 years (range 3–14 years). According to patients' health cards, all had been previously treated with quinine, either parenterally or orally according to Burundi's national policy for treating severe malaria (10 mg/kg 3×/day for 7 days). Clinical and laboratory data are presented in the Table.

**Table:** Clinical data from children at the onset of blackwater fever

Data	Case 1	Case 2	Case 3	Case 4	Case 5	Case 6	Case 7	Case 8	Case 9
Age	13	3	5	14	7	12	14	3	3
Hemoglobin (g/dL)	4.2	3.8	2.2	4.6	5.8	4.3	2.5	2.5	3.1
Parasites/μL	4,700	6,800	8,100	1,680	0	7,600	11,480	5,450	0
Fever*	+	+	+	–	+	+	+	+	+
Jaundice†	+	–	+	+	–	–	–	–	–
Hepatomegaly‡	–	+	–	–	–	–	–	+	+
Splenomegaly‡	–	+	+	–	–	+	+	+	+
Vomiting	–	–	+	+	+	+	–	–	–
Oligoanuria	–	–	–	–	–	–	–	–	–
Abdominal pain	+	–	–	+	–	–	–	+	–

*Axillary temperature >37.5°C. †Total bilirubinemia >1.5 mg/dL. ‡Manually assessed, >2 cm by costal margin.

When BWF occurred, quinine was stopped and artemether (3.2 mg/kg on day 1, then 1.6 mg/kg from day 2 to day 5), was administered intramuscularly in association with 3 days of corticosteroid therapy. All patients had severe anemia requiring blood transfusion according to hospital policy (hemoglobin <4.5 g/dL or <6 g/dL with accompanying dyspnea). Four patients needed l U of blood; 5 other patients needed >1 U. No deaths were recorded, and clinical outcome on discharge was satisfactory: thick smears were negative and hemoglobin levels had improved in all patients.

## Conclusions

In Burundi, chloroquine was replaced by sulfadoxine-pyrimethamine (SP) alone as firstline treatment for uncomplicated malaria in 2001. However, the rapid development of resistance to SP brought back the use of oral quinine, a drug still available in health centers as well as in hospital settings. Since November 2003, artesunate and amodiaquine have replaced SP as firstline treatment in Burundi (13).

The result of the new treatment guidelines was a considerable reduction in the number of hospitalized malaria cases in 2004 (671 cases from January 1 to October 31, 2004). No cases of BWF were observed in this period. Despite changes in policy for the use of firstline antimalarial drugs, however, parenteral quinine continued to be the drug of choice for severe cases throughout this period.

All 9 patients with BWF seen in 2003 (with 1 exception) lived in the area served by Kiremba Hospital and were recorded during an 11-month period. This number represents an incidence of 11.5 cases/100.000 population/year.

In reviewing recent literature, we found only 1 publication on BWF involving an African population (7). The study was carried out at the Dielmo village in Senegal, where 3 cases were detected in a 10-year prospective study in a small population (315 inhabitants). All 3 cases were in children who suffered several malaria attacks and were treated with oral or parenteral quinine, depending on the severity of the case. As a consequence, quinine was withdrawn as the drug of firstline therapy for uncomplicated cases of malaria. No more cases of BWF were recorded during the subsequent 6-year follow-up period.

In our study, patients were all boys admitted to the pediatric ward. No cases of oligoanuria were seen, which is not surprising in pediatric patients (14). At the onset of severe intravascular hemolysis, the blood smears of 2 children were negative for malaria; parasitemia was low in the others. These findings agree with the definition of BWF as being characterized by scanty or absent parasitemia (4,6,9). We were unable to determine G6PD levels in our patients, which is a major limitation of our study. However, in view of the overlap between malaria, quinine administration, and G6PD deficiency, the hemoglobinuria triggered by this deficiency should not be seen as a separate syndrome (10).

The management of our cases included 3 components: First, treatment with parenteral (intramuscular) artemether (3.2 mg/kg on day 1, then 1.6 mg/kg from days 2 to 5) after stopping quinine, according to recent trends in the literature (3–5). Artemisinin derivates have not been implicated in BWF episodes unless given in combination with mefloquine (8). Second, blood transfusion for severe anemia was performed according to the above described hospital policy. And finally, a short course of corticosteroid therapy was administered.

Our experience suggests the need to review the definition of BWF since the syndrome affects not only adult expatriates but also African children. All reported African children with cases of BWF had frequently received oral quinine therapy. African adults seem to be only occasionally affected. This finding suggests that BWF occurs in nonimmune persons or those who have not yet gained immunity. This statement is supported by the lack of cases in adults cured in the same hospital.

To reduce hemolysis, we treated BWF with corticosteroids, even though this step is not recommended by the World Health Organization. Our reasoning was that the phenomenon could be related to immune mechanisms in quinine-sensitized erythrocytes (14).

The influence of quinine seems to be an important factor in the pathogenesis of BWF. Other amino-alcohol drugs such as mefloquine or halofantrine have never been used intensively in Africa, principally because they are expensive. When policy changes lead to less use of oral quinine, BWF syndrome tends to disappear. Further similar reports from other areas in the African continent that would confirm our findings could have important implications on national policies for treating malaria in African children.
